# Association Rule Mining and Network Analysis in Oriental Medicine

**DOI:** 10.1371/journal.pone.0059241

**Published:** 2013-03-15

**Authors:** Dong Hoon Yang, Ji Hoon Kang, Young Bae Park, Young Jae Park, Hwan Sup Oh, Seoung Bum Kim

**Affiliations:** 1 Department of Biofunctional Medicine and Diagnostics, Kyung Hee University, Hoegi-dong, Dongdaemun-gu, Seoul, Republic of Korea; 2 School of Industrial Management Engineering, Korea University, Anam-dong, Seongbuk-gu, Seoul, Republic of Korea; 3 Department of Human Informatics of Oriental Medicine, Interdisciplinary Programs, Kyung Hee University, Giheung-gu, Yongin, Gyeonggi-do, Republic of Korea; 4 Department of Mechanical Engineering, Kyung Hee University, Giheung-gu, Yongin, Gyeonggi-do, Republic of Korea; Dana-Farber Cancer Institute, United States of America

## Abstract

Extracting useful and meaningful patterns from large volumes of text data is of growing importance. In the present study we analyze vast amounts of prescription data, generated from the book of oriental medicine to identify the relationships between the symptoms and the associated medicines used to treat these symptoms. The oriental medicine book used in this study (called Bangyakhappyeon) contains a large number of prescriptions to treat about 54 categorized symptoms and lists the corresponding herbal materials. We used an association rule algorithm combined with network analysis and found useful and informative relationships between the symptoms and medicines.

## Introduction

As a complementary medical system to Western medicine, traditional Korean medicine (TKM) has for thousands of years provided a unique theoretical and practical approach to the treatment of diseases. TKM has been recognized as an effective and safe complementary and alternative medicine because its components are generally extracted from natural products without artificial additives; consequently TKM generally yields mild healing effects with few side effects. In Korea, the scale of the medical service market related to TKM is about 2.7 trillion won and is increasing each year [1]. Recent surveys show that complementary and alternative medicine, including TKM, is widely used in Korea, with usage rates ranging from 29% to 53% among various patient populations. Moreover, the ever-increasing use of Oriental herbal medicine and acupuncture worldwide is a good indication of the public interest in Oriental medicine [2].

Based on traditional theory compiled through thousands of years of practice and research by TKM experts, a large amount of knowledge has accumulated in the form of ancient books and modern literature. The number of prescriptions has increased gradually based on an accumulation of experience with traditional Chinese medicine (TCM) theory. The traditional Chinese Drug Database contains 11,000 herbs, and the Database of Chinese Medical Formulas contains 85,000 prescriptions [3]. Jiang and Li reported a total of 1,554 prescriptions related to spleen-stomach ailments, pointing to the difficulty of selecting a proper prescription [4]. Manually collecting materials and discovering rules on their uses, as is done in current practice, are time-consuming and error-prone. It usually takes several weeks for experts to manually process these documents for further medical tests to verify the effectiveness of a drug for specific symptoms. Moreover, it is becoming harder to understand the interrelated roles of herbal materials in complex prescriptions.

In order to address this problem, data mining algorithms, because of their proven capability to effectively analyze and manage large amounts of data, have been used to uncover useful patterns from documents of Oriental medicine. Data mining is generally defined as the process of extracting meaningful information from large datasets through the use of any relevant data analysis techniques [5], [6]. The techniques in data mining can be utilized to extract meaningful patterns from large volumes of text data and they are called text mining. Unlike conventional data mining tasks that extract the patterns from structured databases, text mining is intended to explore relationship among the objects stored in unstructured database.

Cao *et al*. developed an ontology-based system for extracting knowledge about TCM herbs and formulae from semi-structured text [7]. They developed herb and formula ontologies from seven knowledge sources, including textbooks, codices, encyclopedias, and dictionaries. The two ontologies consist of a set of classes and their relationships and formal axioms for constraining the interpretation of those classes and relationships. Based on the defined ontologies and the canonical description of herb and formula texts, an executable knowledge extraction language was developed that assists in extracting knowledge from the herb and formula texts. The system has been tested on several herb and formula text sources. A knowledge base of more than 2,710 herbs and 5,900 formulas was constructed. The other work regarding the automatic extraction of formula knowledge from the TCM bibliographic literature is the MeDisco/3T system [8]. The MeDisco/3T system iteratively extracts new TCM names and patterns by using a small initial set of formula names to serve as seeds. The MeDisco/3T system is able to correctly extract over 95% of the formula names. Based on the extracted formula names, heuristic rules are used to extract the constituent herb information from the semi-structured abstracts in the literature. With more than 18,000 formulae extracted, the final step is to discover interesting herb pairs and herb family combinations by means of an association rule mining algorithm. Li *et al.* developed the data mining system called TCMiner based on frequent pattern mining and association rule mining [9]. Zhou *et al.* presented an overview of text mining methodologies for TCM [10]. Recently, Hong *et al*. performed frequent analysis to identify relationships between symptoms and prescriptions in TKM [11]. Some studies were conducted to examine the relationship between the herbal materials using an association rule algorithm and network analysis in TCM [12], [13], [14], [15].

Despite the promising results of aforementioned studies, data mining with unstructured data of TCM and TKM is still in its early stages. Clinical practice in oriental medicine is a kind of complex clinical experiments trying to effectively apply a vast amount of largely uncategorized information and data sources concerning symptoms, herbs, and prescriptions. The main purpose of this present study is to investigate the entire Bangyakhappyeon symptom-prescription-drug pattern and analyze the relationships between symptoms and their associated herbal materials. In particular, we focused on identifying herbal materials with a strong association for six main symptoms (cough, overextertion/fatigue, internal damage, aggregation-accumulation, edema, and distension and fullness) and visualizing this association by using network analysis.

### Data

#### Data source – document

**Figure 1:**
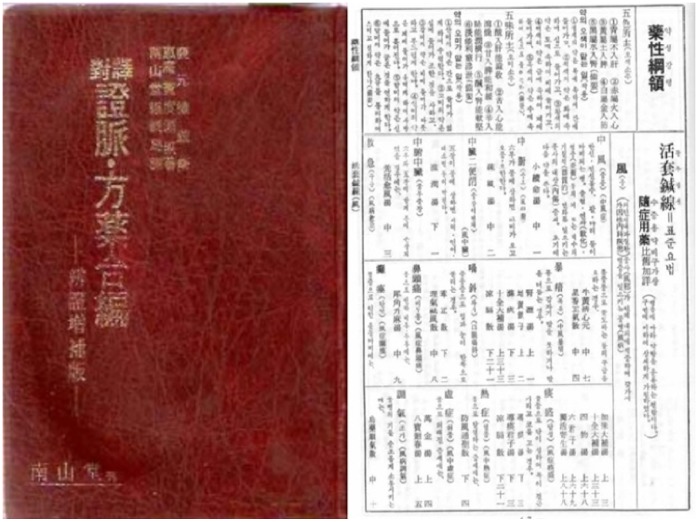
Bangyakhappyeon.

Bangyakhappyeon was written by Hwang Do-Yeon (1807–1884) and his son Hwang Pil-Soo (?–?) in the waning days of the Chosun Dynasty when the country was in a state of social confusion because of the invasion by foreign powers and the initial introduction of Western culture. Figure 1 shows the cover page and one of the content pages of Bangyakhappyeon. Hwang and his son wrote the book in response to this situation in which practical knowledge and solutions took precedence over theological review. Bangyakhappyeon furthered the publication of Asian medical science abstracts in a more compact form, emphasizing a practical perspective. These new publications were a significant counter measure in coping with the inflow of practical Western medicine and enhanced the popularization of TKM. These days, Bangyakhappyeon is widely used by TKM clinicians and highly valued as an indispensable medical prescription manual.

Hwaltuchimsun, a chapter of Bangyakhappyeon consists of 54 categories of main symptoms. Categories 1 through 18 contain miscellaneous frequent diseases, categories 19 through 30 consider the internal parts of the body, including essence, spirit, Qi, and blood. In addition, categories 31 through 52 are devoted to physical maladies such as those of the eyes, ears, and mouth. The remaining two categories (53 through 54) consider gynecology, obstetrics, and pediatrics. Symptoms are summarized based on the cause, nature, and location of the pathological changes at particular stages of diseases. In the present study, among 54 symptoms, we focused on six clinically meaningful symptoms including cough, internal damage, fatigue/overexertion, aggregation-accumulation edema, distention and fullness.

#### Database construction

Bangyakhappyeon contains 521 prescriptions and 305 herbal materials. Most prescriptions (formulas and recipes) comprise several herbal ingredients per prescription. We analyzed the formulas without considering dosages because dosage information in Bangyakhappyeon is highly variable. As shown in Table 1, we constructed a binary matrix in which columns are herbal materials, rows represent prescriptions, and each cell has either a 0 or a 1.

**Table 1:** An example of a database constructed from Bangyakhappyeon.

Materia medica/prescription	Ginseng Radix	Citri Pericarpium	Pinelliae Rhizoma	Poria(red)	…	…
Prescription 1	1	0	0	0		
Prescription 2	1	1	0	1		
Prescription 3	0	0	0	1		
Prescription 4	0	0	1	1		
…						
Prescription 521	1	1	0	0		

Many kinds of prescriptions and herbal materials account for the 54 types of symptoms in the book. If the variety of prescriptions were more extensive, the number of materials would grow in a geometric progression. Therefore, the important medicines for certain symptoms are difficult to discern. The dataset contains several combinations of prescriptions and herbal materials used, including duplications, in 54 symptoms. For example, 63 prescriptions are available for coughs, 91 herbal materials can be used, and 430 materials, including duplications, are available.

## Methods

### Association Rules

Association rules have been widely used to identify relationships between item sets in large databases. Association rules are generated in two stages. First, a set of frequent rules is generated. Second, the strength of the rules, obtained from the first stage is evaluated. For the first stage to generate the rules, an *Apriori* algorithm or a FP-Growth tree has been widely used [16], [17]. Although *Apriori* and *FP-Growth* take different way to identify frequent item sets, the resulting rules are not significantly different [18]. In the present study we adopted an *Apriori* algorithm to discover associated patterns because it is the most well-known association rule induction algorithm [19].

Having found a number of candidate rules from the *Apriori* algorithm, the goal is now to assess the strength of the rules. Three main measures to achieve this goal are support, confidence, and lift. The support value of a rule with an antecedent Item set *A* and a consequent Item set *B* is defined as the proportion of transactions that include all antecedent and consequent item sets. Confidence is defined as the ratio of support value to the number of transactions of all the antecedent items sets. The lift value of a rule is the ratio of the number of transactions of consequent item sets given that antecedent item set has occurred to the number of transactions of consequent item sets in all transactions [20]. A lift value greater than 1 implies that the degree of association between the antecedent and consequent item sets is higher than in a situation in which the antecedent and consequent item sets are independent. In our study symptoms can be considered as an antecedent item set, and the herbal materials can be considered as a consequent item set. To apply an association rule algorithm, we used SPSS Clementine 12.0(www.spss.com).

### Network Analysis

Network analysis provides a nice graphical representation to visualize relationships among the objects in terms of nodes and links. In our analysis, the objects represent symptoms and their associated herbal materials. The graphical display resulting from network analysis enables us to understand the whole relationship among the objects interested. The network can be characterized by the following measures: degree, density, centrality, modularity, and many others [21]. In the present study we used NetMiner 4 (www.netminer.com) to generate a network that visualizes relationships between symptoms and herbal materials.

## Results and Discussion

We used association rules to characterize the relationships between symptoms and herbal materials. Table 2 shows the support, confidence, and lift of the association rules between six main symptoms (antecedent) and their associated herbal materials (consequent) that have a confidence value of at least 20%. The minimum confidence value is usually determined by the user.

**Table 2:** Support, confidence, and lift between six symptoms and their associated herbal materials.

Antecedent (symptom)	Consequent (herbal materials)	Support (%)	Confidence (%)	Lift
Coughs	Pinelliae Rhizoma	4.2	46.0	2.2
	Citri Pericarpium	3.9	42.9	1.3
	Rehmanniae Radix Preparat	2.2	23.8	1.8
	Poria(red)	2.0	22.2	1.3
	Poria(white)	2.0	22.2	1.1
	Armeniacae Semen	1.9	20.6	3.0
Overexertion/Fatigue	Rehmanniae Radix Preparat	3.5	70.5	4.5
	Angelicae Gigantis Radix	3.3	67.6	1.9
	Ginseng Radix	2.0	41.1	1.4
	Poria(white)	1.9	38.2	1.9
	Dioscoreae Rhizoma	1.7	35.3	5.0
	Paeoniae Radix Alba	1.7	35.3	1.8
	Atractylodis Rhizoma Alba	1.6	32.3	1.2
	Corni Fructus	1.4	29.4	5.8
	Capreoli Cornu	1.3	26.5	8.2
	Astragali Radix	1.3	26.5	2.4
	Achyranthis Radix	1.2	23.5	5.0
	Pulvis Aconiti Tuberis Purificatum	1.2	23.5	2.8
	Cinnamomi Cortex Spissus	1.2	23.5	1.8
	Moutan Cortex	1.0	20.6	3.6
	Schizandrae Fructus	1.0	20.6	3.4
	Alismatis Rhizoma	1.0	20.6	2.0
Internal Damage	Citri Pericarpium	4.1	71.8	2.1
	Atractylodis Rhizoma Alba	3.0	53.8	1.9
	Poria(white)	2.3	41.0	2.0
	Magnoliae Cortex	2.3	41.0	2.8
	Ginseng Radix	2.3	41.0	1.4
	Pinelliae Rhizoma	2.2	38.5	1.9
	Aaurantii Immaturus Fructus	1.9	33.3	4.4
	Cyperi Rhizoma	1.7	30.8	2.8
	Atractylodis Rhizoma	1.7	30.8	2.2
	Massa Medicata Fermentata	1.6	28.2	4.3
	Aucklandiae Radix	1.3	23.1	2.1
	Amomi Fuctus	1.3	23.1	2.9
	Paeoniae Radix Alba	1.2	20.5	1.1
	Crataegii Fructus	1.2	20.5	5.7
Aggregation-Acumulation	Citri Pericarpium	1.6	78.6	2.4
	Atractylodis Rhizoma	1.0	50.0	3.7
	Magnoliae Cortex	0.9	42.9	3.1
	Cyperi Rhizoma	0.7	35.7	3.5
	Poria(red)	0.7	35.7	2.1
	Pinelliae Rhizoma	0.6	28.6	1.5
	Atractylodis Rhizoma Alba	0.6	28.6	1.1
	Massa Medicata Fermentata	0.4	21.4	4.0
	Citrii Unshiu Immaturi Pericarpium	0.4	21.4	3.6
	Zingiberis Rhizoma Siccus	0.4	21.4	2.3
	Ponciri Fructus Pericarpium	0.4	21.4	2.0
Edema	Atractylodis Rhizoma Alba	1.2	66.7	2.4
	Alismatis Rhizoma	0.9	50.0	4.7
	Poria(red)	0.9	50.0	2.8
	Citri Pericarpium	0.9	50.0	1.6
	Magnoliae Cortex	0.7	41.7	3.1
	Ginseng Radix	0.6	33.3	1.2
	Mori Radicis Cortex	0.4	25.0	7.8
	Polyporus	0.4	25.0	5.1
	Agastachis Herba	0.4	25.0	5.1
	Arecae Semen	0.4	25.0	4.3
	Pulvis Aconiti Tuberis Purificatum	0.4	25.0	3.3
	Zingiberis Rhizoma Siccus	0.4	25.0	2.7
	Aucklandiae Radix	0.4	25.0	2.4
	Atractylodis Rhizoma	0.4	25.0	2.0
	Pinelliae Rhizoma	0.4	25.0	1.3
	Poria(white)	0.4	25.0	1.3
Distension and Fullness	Magnoliae Cortex	0.7	71.4	5.2
	Citrii Unshiu Immaturi Pericarpium	0.6	57.1	9.1
	Aucklandiae Radix	0.6	57.1	5.3
	Pinelliae Rhizoma	0.6	57.1	3.0
	Ginseng Radix	0.6	57.1	2.0
	Citri Pericarpium	0.6	57.1	1.8
	Alpiniae Fructus	0.4	42.9	15.1
	Perilla Herba	0.4	42.9	5.8
	Angelicae Gigantis Radix	0.4	42.9	1.3
	Amomi Globosi Semen	0.3	28.6	16.8
	Zedoariae Rhizoma	0.3	28.6	12.6
	Arecae Pericarpium	0.3	28.6	10.8
	Evodiae Fructus	0.3	28.6	8.9
	Akebiae Caulis	0.3	28.6	6.0
	Arecae Semen	0.3	28.6	5.0
	Amomi Fuctus	0.3	28.6	4.0
	Cimicifugae Rhizoma	0.3	28.6	3.2
	Zingiberis Rhizoma Siccus	0.3	28.6	3.1
	Cyperi Rhizoma	0.3	28.6	2.9
	Alismatis Rhizoma	0.3	28.6	2.8
	Bupleuri Radix	0.3	28.6	2.8
	Ponciri Fructus Pericarpium	0.3	28.6	2.7
	Poria(red)	0.3	28.6	1.7
	Poria(white)	0.3	28.6	1.5
	Ligustici Rhizoma	0.3	28.6	1.3
	Atractylodis Rhizoma Alba	0.3	28.6	1.1

As mentioned earlier, the support of a rule is simply a percentage of occurrences that include both the antecedent (symptom) and consequent (herbal material) sets. The values of confidence and lift can be used to judge the strength of rules. Herbal materials with high confidence and lift values have strong relationships with the symptoms. For example, for cough, the rule (cough *→* Citri Pericarpium) has the highest confidence, meaning that Citri Pericarpium is the most frequently used herbal material for treating coughs. However, it is interesting to note that the rule (cough *→* Citri Pericarpium) has a relatively low lift value (1.3). This implies that Citri Pericarpium is frequently used to treat other symptoms as well as coughs, and thus, can be considered as a generally used material. Conversely, Armeniacae Semen, despite its relatively lower confidence, has a high lift value. This implies that Armeniacae Semen is an herbal material specifically used for treating coughs.

As for overexertion/fatigue, the rules (overexertion/fatigue *→* Rehmanniae Radix Preparat, overexertion/fatigue *→* Angelicae Gigantis Radix) have high confidence, implying that those herbal materials are commonly used to treat overexertion/fatigue. Some rules (overexertion/fatigue *→* Capreoli Cornu, overexertion/fatigue *→* Corni Fructus) have the high lift and relatively low confidence, meaning that Capreoli Cornu and Corni Fructus are specified herbal materials for the prescription of overexertion/fatigue. It is interesting to note that the rule (overexertion/fatigue *→* Achyranthis Radix) has a high lift value. This may be because Achyranthis Radix is generally adopted for treating back pain and knee pain in TKM.

The important rules for other symptoms (internal damage, aggregation-accumulation, edema, and distension and fullness) in Table 2 can be summarized as follows. Internal damage and aggregation-accumulation share the same herbal materials such as Citri Pericarpium, Atractylodis Rhizoma, and Magnoliae Cortex. Those herbal materials are well known for relieving the stagnation of Qi, promoting digestion, and removing food stagnation. An interesting result can be found in the analysis of distension and fullness. Alpiniae Fructus (the second highest lift value) has been used to treat seminal emission with Yang-warming and kidney-tonifying effects. This high relationship between Alpiniae Fructus and distension and fullness is a somewhat new for clinicians. It may be worthwhile to further investigate the effect of Alpiniae Fructus for distension and fullness.

**Figure 2:**
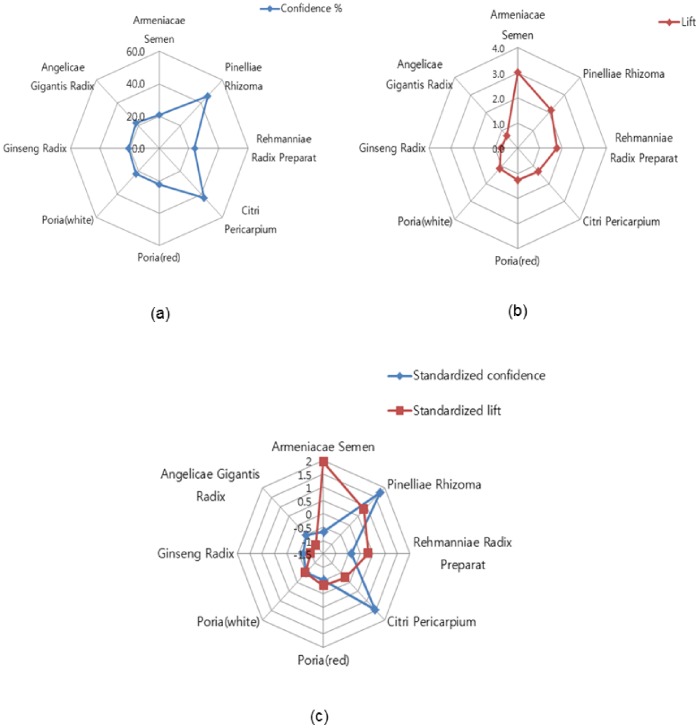
Radar charts of (a) confidence, (b) lift, and (c) standardized confidence and lift values for association rules between coughs and their associated herbal materials.

The results in Table 2 can be visualized by the radar charts shown in Figure 2. The individual charts for confidence (Figure 2 (a)) and lift (Figure 2(b)) show which of the herbal materials have high values of confidence and lift for coughs. Figure 2 (c) shows the values of confidence and lift simultaneously by using their standardized values.

It can be seen that Armeniacae Semen and Rehmanniae Radix Preparat have larger lift values compared with their confidence values; the opposite is true for Pinelliae Rhizoma and Citri Pericarpium. As mentioned earlier, the larger lift values (compared with their confidence values) of Armeniacae Semen and Rehmanniae Radix Preparat imply that these medicines are preferred for treating coughs. On the other hand, the low lift (compared with confidence values) values of Pinelliae Rhizoma and Citri Pericarpium imply that they are globally used medicines for general symptoms, including coughs. A cough has two main symptoms, tussis and phlegm. The TKM indicates that Pinelliae Rhizoma and Citri Pericarpium are preferred for treatment of these symptoms. As for Armeniace Semen, it is known as a medicine especially used for treating coughs because it must be used cautiously in mixtures with other medicines. Association rules between symptoms and herbal materials obtained in the present study were corroborated by consulting TKM clinicians.

**Figure 3:**
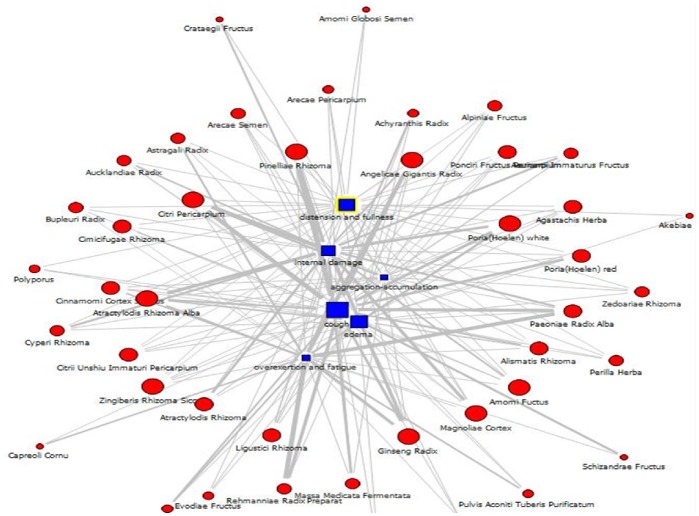
A network graph to visualize association rules between symptoms (cough, overextertion/fatigue, internal damage, aggregation-accumulation, edema, and distension and fullness) and their associated herbal materials.

Figure 3 displays the result of network analysis to illustrate the relationships between six main symptoms and their associated herbal materials. The size of squares (symptoms) and circles (herbal materials) represents the frequency of elements with the prescriptions, and the thickness of lines indicates the strength of association rules. For example, Ginseng Radix, represented by a large circle, has a high frequency as treatment for all six symptoms considered here. It has been recognized in TKM that Ginseng Radix is a commonly used and multipurpose herbal material.

**Figure 4:**
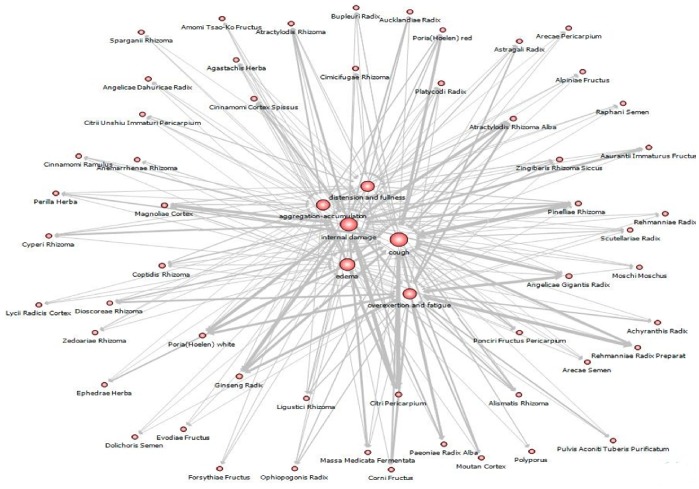
A network that visualizes the degree centrality of symptoms (internal damage, edema, coughs, aggregation-accumulation, distension and fullness, and overexertion and fatigue) and their related herbal materials.

Figure 4 shows the network that represents the relationship between six main symptoms and the herbal materials in terms of the degree centrality, one of the common measures in network analysis. The degree centrality is defined as the number of links that a node has. Table 3 summarizes the values of degree centrality of six symptoms appeared in Figure 4. We see from Table 3 that cough has the highest degree centrality among six symptoms, implying that many types of herbal materials can be used to treat coughs.

**Table 3:** Degree centrality of six symptoms appeared in Figure 4.

Symptoms	Degree Centrality
Cough	0.793
Internal Damage	0.776
Edema	0.672
Aggregation-Accumulation	0.569
Distension and Fullness	0.569
Overexertion and Fatigue	0.552

Through network analysis, we can readily see the list of herbal materials that were used together to treat a certain symptom. Overall, network analysis is very helpful in understanding the fundamental principles of prescriptions and the effects of medicines in TKM.

### Conclusions

This paper aims at extracting useful information from TKM databases. We have used association rules and a couple of graphical approaches to reveal the patterns associated with symptoms and the related herbal materials. Association analysis can help us deduce meaningful rules on associations among item sets. Support, confidence, and lift, calculated from the association rules can be used to assess the strength of these rules. In addition, we used radar charts and network analysis to effectively visualize the association rules. Association rules between disease symptoms and herbal materials can be useful for the development of new medicines in TKM because they help identify the important herbs which correspond to certain prescriptions. Moreover, when a clinician diagnoses and treats patients, full use can be made of the information to arrive at an accurate diagnosis and effective treatment. The analysis procedure presented in this paper can be applied to other publications or fields (such as acupuncture and moxibustion) in TKM to summarize the documents and to uncover useful knowledge on human health. We hope that the present study increases awareness within the TKM community of efficient methodologies to improve diagnosis in TKM treatment.
